# Small RNAs, spermatogenesis, and male infertility: a decade of retrospect

**DOI:** 10.1186/s12958-023-01155-w

**Published:** 2023-11-04

**Authors:** Meghali Joshi, Shruti Sethi, Poonam Mehta, Anamika Kumari, Singh Rajender

**Affiliations:** 1 Division of Endocrinology, Central Drug Research Institute, Lucknow, Uttar Pradesh India; 2 Academy of Scientific and Innovative Research (AcSIR), Ghaziabad, Uttar Pradesh India

**Keywords:** Spermatogenesis, miRNA, Small RNAs, Non-coding RNA, piRNA, tsRNA, Endogenous siRNA

## Abstract

Small non-coding RNAs (sncRNAs), being the top regulators of gene expression, have been thoroughly studied in various biological systems, including the testis. Research over the last decade has generated significant evidence in support of the crucial roles of sncRNAs in male reproduction, particularly in the maintenance of primordial germ cells, meiosis, spermiogenesis, sperm fertility, and early post-fertilization development. The most commonly studied small RNAs in spermatogenesis are microRNAs (miRNAs), PIWI-interacting RNA (piRNA), small interfering RNA (siRNA), and transfer RNA-derived small RNAs (ts-RNAs). Small non-coding RNAs are crucial in regulating the dynamic, spatial, and temporal gene expression profiles in developing germ cells. A number of small RNAs, particularly miRNAs and tsRNAs, are loaded on spermatozoa during their epididymal maturation. With regard to their roles in fertility, miRNAs have been studied most often, followed by piRNAs and tsRNAs. Dysregulation of more than 100 miRNAs has been shown to correlate with infertility. piRNA and tsRNA dysregulations in infertility have been studied in only 3–5 studies. Sperm-borne small RNAs hold great potential to act as biomarkers of sperm quality and fertility. In this article, we review the role of small RNAs in spermatogenesis, their association with infertility, and their potential as biomarkers of sperm quality and fertility.

## Background

Spermatogenesis is a highly orchestrated process under multilayered regulation of gene expression that maintains the balance between cell proliferation and differentiation to ensure incessant production of spermatozoa from the spermatogonial stem cells by a series of transformational events, including mitosis, meiosis and cell differentiation [1, 2]. Changes in the gene expression affect the risk of infertility by altering critical signaling pathways in the testis. Moreover, changes in gene sequence were considered to be the most important regulators of gene expression, founding the basis of a number of studies on gene polymorphisms [3]. Eventually, the importance of epigenetic regulators was realized and methylation analysis of genes critical to spermatogenesis dominated the field for about a decade [4]. The identification of small RNAs as gene expression regulators has added another dimension to the theory of gene expression regulation [5]. In the last more than one decade, massive progress in this area has generated a plethora of data on the role of small RNAs in gene regulation and the risk of a number of associated human diseases [6]. Studies on animal models and human male infertility have identified a significant number of small RNAs that play crucial roles in the regulation of spermatogenesis [7].

Earlier, RT-PCR was used to analyze the expression of small RNAs associated with infertility [8, 9]. The availability of miRNA microarrays promised quick scanning of the changes in miRNA levels in human diseases [10]. The advent of next generation sequencing technology promised much beyond the microarrays, i.e. apart from known candidates, sequencing approach could also identify new miRNAs that correlate with infertility [11, 12]. Studies on testicular germ cells have shown that small RNAs undergo significant changes in their level and patterns of expression during spermatogenic development of the germ cells [13]. Germ cells (spermatocytes, round spermatids and spermatozoa) share a large number of small non-coding RNAs apart from having a set of RNAs unique to each cell type [14]. This suggested that the regulation of spermatogenesis is more complicated than understood till now. For these to take shape, we evaluate and review the role of small non-coding RNAs in spermatogenesis and correlation of their altered expressions with male infertility.

## The small RNA family

Non-coding RNAs consist of different classes based on their size, biogenesis, and functions. Small non-coding RNAs are smaller than 200 nucleotides and comprise of housekeeping sncRNAs, such as small nucleolar RNAs (snoRNAs) and small nuclear RNAs (snRNAs), and regulatory sncRNAs, such as miRNAs, endogeneous siRNAs, Piwi RNAs, tsRNAs (tRNA derived small RNAs), and rsRNAs (rRNA derived small RNAs). Several studies have been conducted to identify sncRNAs signature of male infertility using techniques like qRT-PCR, microarray, and next generation sequencing. Among all small non-coding RNAs, the most commonly investigated are miRNAs, piRNAs and endogenous siRNAs. Recently, the emphasis has shifted to other sncRNAs, such as tRNA derived small RNAs (tsRNAs) and rRNA derived small RNAs (rsRNAs). Increasing evidences suggest that miRNAs are very crucial in germ cell development and early embryonic development [15].

Like miRNAs, siRNAs are double stranded small ncRNAs but siRNAs require Dicer for processing whereas miRNAs are processed by both Drosha and Dicer. After processing, siRNAs are loaded onto Ago2 protein, which mediates the cleavage of target mRNA. Endo-siRNAs were first reported in yeast and later in plants [16]. In mammals, endo-siRNAs were first reported in murine spermatogenic cells [17]. siRNAs have been used in research as a tool to knock-down a particular gene in a bid to identify its function [7]. In *Drosophila melanogaster* and mice, diverse roles of endo-siRNAs, including post-transcriptional gene regulation, transposable elements regulation, and modification of DNA by DNA methylation, have been reported [18]. A lone study reported reduced early embryonic development when sperm with altered miRNAs and endo-siRNAs profiles were injected into wild type eggs by ICSI, which was later rescued by injecting wild type sperm derived sncRNAs into embryos [19], suggesting that endo-siRNAs might play role in early embryonic development.

Other class of small non-coding RNAs is piRNA (25–32 nucleotide), which interacts with PIWI protein (a subclade of AGO proteins) and silence transposons in germ cells, epigenetically and post-transcriptionally [20–24]. PIWI proteins have been largely studied in drosophila and have roles in maintaining germ line stem cells by regulating germ cell division [25]. In male germ cells, piRNAs appear in two distinct waves: one in the primordial germ cells (PGCs), called as pre-pachytene piRNAs and the other during the meiotic prophase, called as pachytene piRNAs [26]. The pre-pachytene piRNAs due to their repeat derived nature are known to regulate de novo methylation and transposable elements (TE) expression during the methylation re-writing phase of the embryonic development [27], while the pachytene piRNAs have roles beyond silencing of the transposable elements. Pachytene piRNAs have been shown to target mRNA and lncRNAs via PTGS (Post transcriptional gene silencing) mechanism [28]. Altered expressions of sperm piRNAs were found to be associated with sperm concentration and fertilization rate after ICSI, indicating that sperm piRNAs are important for fertilization.

Another class of small non-coding RNAs are tRNA derived small RNAs (tsRNAs), which have been recently studied in the male germ cells. Human sperm is abundantly rich in tsRNAs and comprise of approximately 56% of the total small non-coding RNAs [29]. In epididymis, both miRNAs and tsRNAs are loaded onto spermatozoa via epididymosomes during sperm maturation, suggesting that tRNA fragments could have key roles in fertilization and embryonic development [29]. Further, tRNA-derived small RNAs have been majorly found to be involved in intergenerational and transgenerational/epigenetic inheritance of environmental experiences/stressors and metabolic disorder [30, 31].

Since the discovery of small non-coding RNAs in spermatozoa, it has become very clear that most of the genes in mammals are regulated by sncRNAs [32, 33]. We reviewed the importance of small RNAs in germ cells, testis and male infertility about a decade ago [4]. This field of small RNAs in spermatogenesis, male fertility and transgenerational inheritance has expanded tremendously in the last one decade. Therefore, the aim of this review is to update the field of small RNAs in spermatogenesis and male infertility with a particular emphasis on their potential to act as biomarkers of sperm fertility and quality.

### miRNAs

MicroRNAs were first identified in 1993 when researchers studying the development of *Caenorhabditis elegans* discovered a short non-coding RNA. Since then, they have been discovered in almost all organisms and various studies have been conducted to elucidate their roles in germ cells. miRNAs are short nucleotide structures (∼22nt), which regulate gene function at the post-transcriptional level by destabilizing the target mRNA molecules [25, 34]. miRNAs regulate the expression of protein-coding genes in all germ cells.

## Generation of miRNAs

**Fig. 1:**
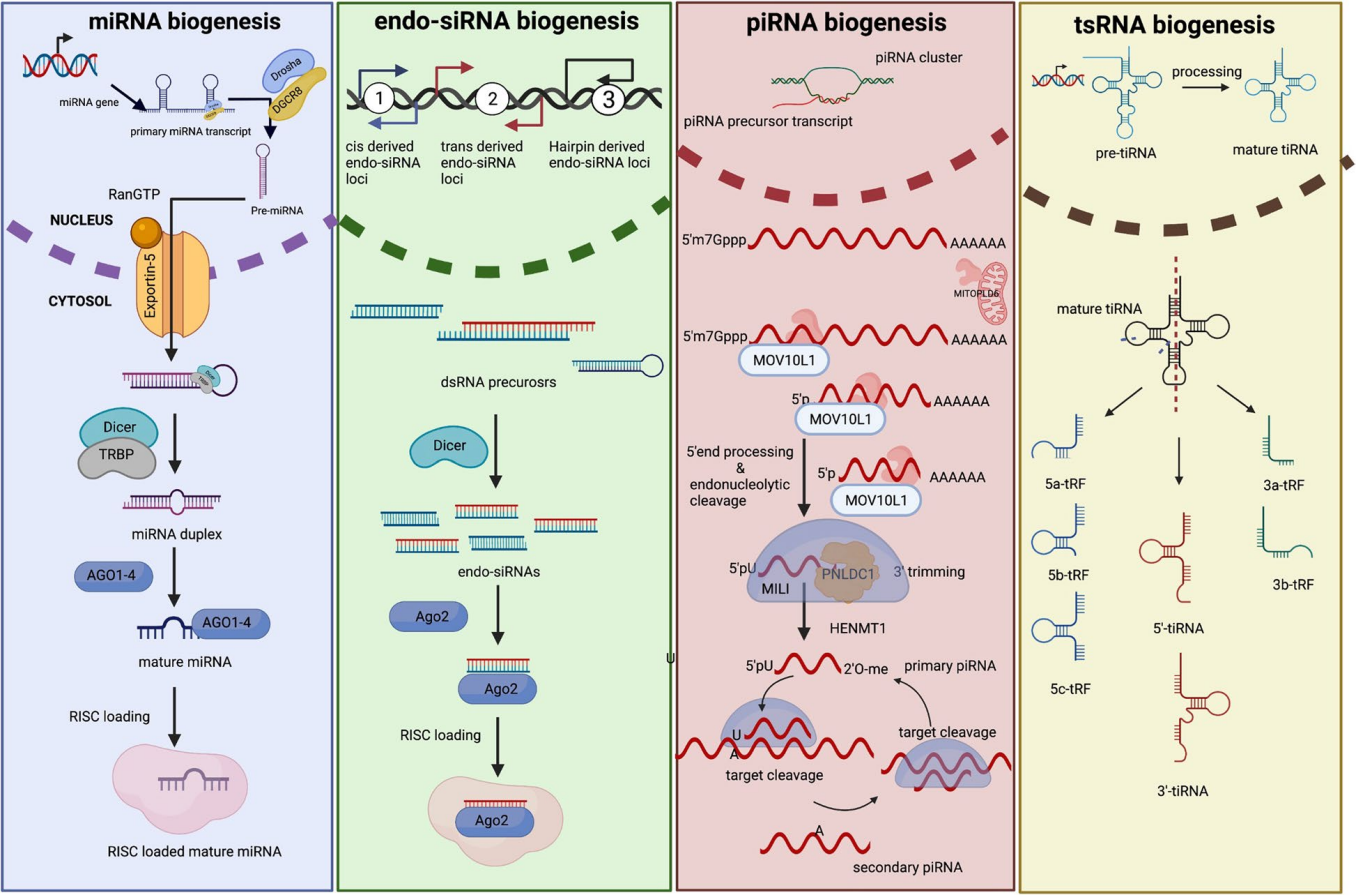
Biogenesis of small non-coding RNAs

The biogenesis of miRNA initiates from the nucleus where miRNA genes get transcribed into long hairpin primary transcripts. Drosha along with its cofactor DGCR8 forms a complex called microprocessor and processes primary transcripts into small hairpin RNAs (pre-miRNA) (Fig. 1). Following Drosha processing, pre-miRNA is transported to the cytoplasm for further processing through a protein, Exportin-5. In the cytoplasm, pre-miRNA is cleaved by another protein called Dicer to yield a small RNA duplex. This small RNA duplex is loaded onto an AGO protein, forming a complex called RNA-induced silencing complex (RISC), which unwinds the RNA. The unwinding of the duplex generates two strands, passenger strand (miRNA*) and guide strand. miRNAs can target multiple mRNA molecules in a specific manner, which makes miRNAs powerful regulatory molecules [35].

### miRNAs in PGCs

In the primordial germ cells (PGCs), miRNAs regulate a number of pluripotency genes involved in germ cells specification. In human embryonic stem (ES) cells, miR-145 completely suppresses the expression of OCT4 and partially suppresses SOX2 expression and hence promotes differentiation. The deletion of Drosha and Dicer in male PGCs disrupted its propagation, differentiation, and maturation [36]. PGCs start to differentiate from embryonic stages E11.5 stage and at E13.5 female PGCs enter meiosis while male PGCs enter mitotic arrest in mice. It has been reported that several miRNAs play an important role in gonad sexual determination. It was reported that expression of miR-103-3p, let-7 g-5p, miR-107-3p, and miR-26a-5p was down-regulated in E11.5 male somatic cells [37]. These miRNAs target key genes (Cyp26b1, Fgf9) in male germ cell differentiation. Moreover, eighteen miRNAs (mmu-miR-19a-3p, mmu-miR-22-3p, mmu-miR-30a-5p, mmu-miR-30d-5p, mmu-miR-30e-5p, mmu-miR-125a-3p, mmu-miR-139-5p, mmu-miR-140-5p, mmu-miR-149-5p, mmu-miR-185-5p, mmu-miR-202-3p, mmu-miR-204-5p, mmu-miR-214-3p, mmu-miR-500-3p, mmu-miR-532-3p, mmu-miR-532-5p, mmu-miR-665-3p, mmu-miR-667-3p) were up-regulated in E13.5 male somatic cells, which target genes related to activin signaling pathway, and activin is known to inhibit Cyp26B1 [37]. They also found two clusters of miRNAs (miR-199-214, miR-182-183-96) to be differentially expressed in embryonic stages E11.5, E12.5 and E13.5, whose targets are involved in gonad development [37]. Male germline committed PGCs showed up-regulation of let-7 family, miR-125a, and miR-9 [36], whereas miR-29b was found to be up-regulated in female germ line committed PGCs, where it epigenetically regulates gene expression by targeting Dnmt3a and Dnmt3b [38].

### miRNAs in spermatogenesis

Spermatogenesis is the main and crucial step of male reproduction, which requires an accurately, spatially and temporally regulated miRNA expression pattern [39]. To maintain the germ cells population throughout sexual maturity, the spermatogonial stem cells (SSCs) must divide in order to self-renew the stem cell pool. Through high-throughput sequencing, it was revealed that a number of miRNAs contribute to the regulation of SSC status. Recently, many studies investigating the role of small RNAs in spermatogenesis have revealed the importance of miRNAs during this process. Zhou et al. [40] reported that miR-663a is important for the proliferation of SSCs [40]. They found NFIX to be the direct target of miR-663a and that silencing of miR-663a leads to an increase in the proliferation rate and late apoptosis of human SSCs in-vitro [40]. In another study, miRNA functional assays suggested that miR-322 is critical for SSC self-renewal by targeting RASSF8 (ras association domain family 8) and miR-100 promotes the proliferation of spermatogonial stem cells via regulating Stat3 [41, 42]. The fate of SSCs is mainly determined by genetic and epigenetic factors. Similarly, molecular studies on the SSCs revealed that miRNA-31-5p regulates the proliferation, DNA synthesis, and apoptosis of human SSCs in association with PAK1-JAZF1-cyclin A2 pathway [43]. Liu et al. [44] demonstrated that Chd1 (chromo domain helicase/ATPase DNA binding protein 1-like) mediated downregulation of miR-486 promotes self-renewal potential of SSCs via MMP2 (matrix metalloproteinase 2) regulation [44].

Tan et al. [11] undertook next-generation sequencing to compare small RNA signatures between different cell populations like SSC, Sertoli cells, developing germ cells, and mouse embryonic stem cells grown in vitro [11]. It was concluded that miRNA and endo-siRNA expression levels decline as spermatogenesis proceeds while piRNA expression elevates. Apart from finding several novel miRNAs expressed in mouse SSCs, the group also reported miRNAs common between the SSCs and embryonic stem cells (ESCs) in order to understand the molecular mechanism involved in the conversion of SSCs to ESCs known as germline-derived pluripotent stem cells [11]. In another sequencing study, miRNA expression profiling was conducted in Thy1^+^ cell population, which is highly enriched in SSCs, and Thy1^−^ cell population, composed mainly of testicular somatic cells. After comparing the two groups, they found that miR-21, miR-34c, miR-182, miR-183, and miR-146a were preferentially expressed in the Thy1^+^ cell population compared with Thy1^−^ somatic cells [45]. They further demonstrated that the inhibition of miR-21 in SSC-enriched germ cell culture increased the rate of apoptosis in germ cells; therefore, it is critical for the survival of SSC population [45]. Despite these advancements, the functions and mechanisms of a number of miRNAs in regulating stem cell renewal and differentiation are yet to be explored. He et al. [46] reported that miRNA-20 and miRNA-106a were preferentially expressed in mouse SSCs. In-silico miRNA target prediction reported, STAT3 and CCND1 as the direct targets of miRNA-20 and miRNA-106a. Furthermore, the knockdown of Stat3 and Ccnd1 lead to SSC renewal [46]. Similarly, in another study, it was reported that miR-202 is the key regulatory miRNA for SSCs renewal and maintenance. The function of miR-202 was exploited by knockdown through CRISPR-Cas9 technology and it was found that the absence of miR-202 could lead to early differentiation, reduced stem cell property, and increased mitosis and apoptosis [47]. Through iTRAQ-based proteomic analysis and RNA sequencing, the target genes were identified, which were mainly cell cycle regulators and RNA binding proteins. It was further reported that miR-202 targets Rbfox2 and cpeb1, which are responsible for the differentiation of SSCs into meiotic cells [47].

miR-122a, miR-18a, and miR-34c are some of the crucial miRNAs regulating pivotal steps in spermatogenesis [48]. In the male germ cells, miR-122a is expressed in the late-stage germ cells and it down-regulates the expression of transition protein 2 (Tnp2) post-transcriptionally, a protein involved in replacing histones during chromatin condensation [48]. miR-18a belongs to the miR-17-92 cluster and is associated with the regulation of genes involved in cancer [49]. miR-18a is an important miRNA for spermatogenesis as it directly targets heat shock factor 2 (*Hsf2*), a transcription factor required for the regulation of many genes involved in spermatogenesis and embryogenesis [48, 50]. miR-17-92 cluster consists of miRNAs from four different families (miR-17, miR-18, miR-19, and miR-25). Knockout studies on mir-17-92 cluster have shown reduced testis weight and size and some seminiferous tubules with only Sertoli cells, but the mice were fertile [51]. The above-mentioned targeted disruption of miR-17-92 in the testes of adult mice gives rise to severe testicular degeneration, empty tubules and low sperm production. This phenotype is due to the reduced number of spermatogonia and spermatogonial stem cells (SSCs), and significantly increased germ cell apoptosis in the testes of miR-17-92-deficient mice. All the above suggest that miR-17-92 is essential for normal spermatogenesis in mice [52]. Recently, a study has shown a significantly reduced fertility in miR-17 ~ 92^+/−^ and miR-106b-25^−/−^ double mutant male mice due to oligozoospermia and disrupted spermatogenesis [53]. In another study, miR-469 was found to be up-regulated in GRTH/DDX25 null mice where it was reported to target Tnp2 and Prm2, which ultimately resulted in infertility due to failure in the generation of elongated spermatids [54]. Recently, X-linked miRNAs were evaluated in highly purified spermatogenic cells at different stages, finding that X-linked spermatogenesis-related miRNAs (SpermiRs) expressed in early meiotic phase and showed a conserve testis specific expression in mammals [55]. This study revealed the compensatory upregulation of miR-465a-5p, after knockdown of another abundant miRNA (miR-741) in cultured mouse SSCs, which did not affect the genome-wide mRNA expression levels in cell line. Ota et al. [56] found that X-linked miR-871 and miR-880 cooperatively regulate spermatogenesis via the WNT/β-catenin pathway during testicular germ cell development by targeting Fzd4 (WNT receptor), which further indicates that WNT/β-catenin signaling promotes proliferation of SSCs, but represses their differentiation. Double mutant mice having deletion covering miR-871 and miR-880 resulted in meiotic arrest in a few seminiferous tubules [56].

In a recent study, miR-202-5p was found to be critical to spermatogenesis as its in-vivo inhibition showed spermatogenic arrest in murine testis seminiferous tubules [57]. MiR-34 family comprises of six members (miR-34a, miR-34b, miR-34c, miR-449a, miR-449b, miR-449c) located on three different chromosomes 1p36.22, 11q23.1, and 5q11.2. It has been reported that miR-34 family has significant role in spermatogenesis owing to its high expression in the male germ cells [58, 59]. The members of this family are expressed in spermatogonial stem cells (SSCs) and as well as spermatozoa. The expressions of these family members shoot up in the beginning of meiosis, suggesting their role in spermatocytes [60]. The miR-34 family mainly targets the genes of cell cycle, such as Notch1, Cdk4, and Myc [61]. Interestingly, disruption of a few members of this family led to an increase in the expression of other members showing compensatory effects [58]. Wu et al. [59] found that miR-34b/c and miR-449 clusters are functionally redundant. They inactivated both the clusters simultaneously and found disruption of their target genes involved in spermatogenesis, brain development, and microtubule dynamics. MiR-34c is also present in the pachytene spermatocytes and round spermatids [59]. Tgif2 and Notch are the two transcription factors important for spermatogenesis that are direct targets of miR-34c [61]. MiR-34c along with other miRNAs, such as miR-449 and 34b lead to apoptosis of germ cells by targeting BCL2 and ATF1 [62, 63].

### miRNAs in male infertility

**Fig. 2:**
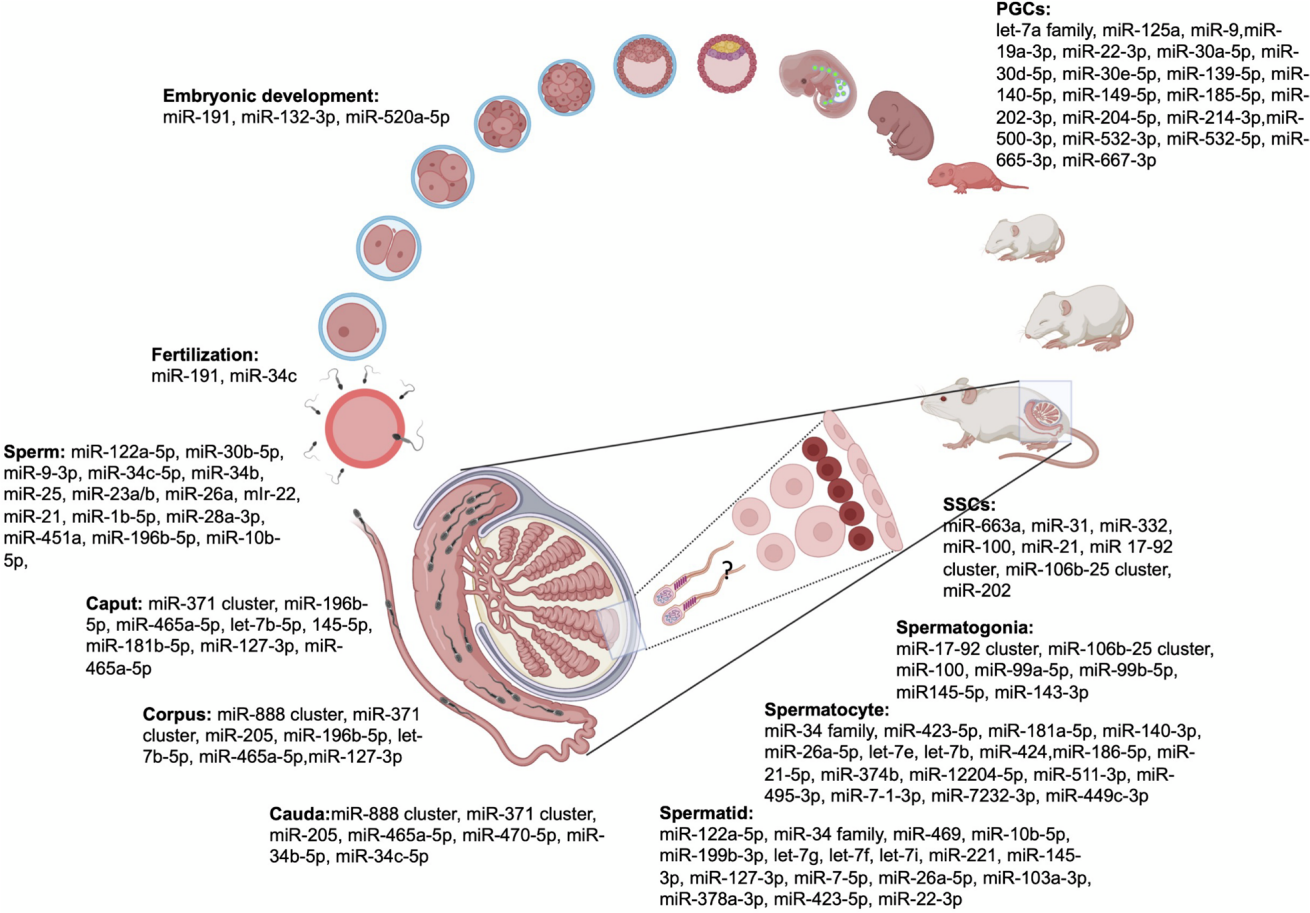
miRNAs critical to various stages of germ cell development in rodents. ?- represents the lack of study on testicular sperm miRNAs

Since miRNAs play critical roles in gene regulation during spermatogenesis, their alterations could lead to male infertility. It is interesting to note that semen contains extracellular and intracellular small RNAs. miRNAs have been thoroughly investigating to have a significant link to numerous facets of male infertility (Table 1). The intracellular small RNAs might have originated in the testis during spermatogenesis or during their maturation in the epididymis. Spermatozoa are well known to lose and gain small RNAs during their transit through epididymis. Apart from intracellular small RNAs, seminal fluid has cell free small RNAs, which might have originated in the testis, epididymis or in male accessory glands. Both the intracellular and cell free small RNAs have been investigated for their roles in fertility and their correlation with infertility. In the following sections, we will discuss testicular miRNAs, sperm-borne miRNA, epididymal small RNAs to highlight their importance in spermatogenesis, fertility and infertility (Fig. 2).

**Table 1:** Studies on differential expression of miRNAs in male infertility

**Study**	**Population type/size**	**Sample**	**Methodology**	**Inference**
Halima et al. [66]	12 SCOS (Sertoli cell only syndrome), 12 MA (Mixed atrophy), 16 GA (Germ cell arrest) patients and 16 NC (Normal Control)	Testicular biopsy	Microarray	About 197, 68, and 46 differentially expressed miRNAs were found in SCOS (Sertoli cell only syndrome), MA (Mixed atrophy), GA (Germ cell arrest) patients, respectively as compared to controls. hsa-miR-34b*, hsa-miR-34b, hsa-miR-34c-5p, and hsa-miR-449a were highly upregulated in SCOS, MA, GA patients compared with fertile control.
Lian et al. [65]	3 non-obstructive azoospermic and 2 fertile controls	Testicular biopsy	Microarray	A total of 154 differentially down-regulated and 19 up-regulated miRNAs in non-obstructive azoospermic patients compared with fertile controls. Differential expression of miR-302a, miR-491-3p, miR-520d-3p and miR-383 was confirmed by qRT-PCR.
Noveski et al. [68]	27 hypospermatogenesis, 3 hypospermatogenesis with AZFc deletion, 8 SCOS and 2 MA, 6 normal spermatogenesis	Testicular biopsy	Microarray	A total of 58 differentially expressed miRNAs. miR-34b, mir-449b, mir-517c were downregulated in all studied groups compared with control.
Zhang et al. [69]	13 non-obstructive azoospermic and 6 fertile controls	Testicular biopsy	Microarray	One hundred twenty-nine miRNAs were found to be differentially expressed in testicular tissues of non-obstructive azoospermic patients as compared to controls. Combination of two miRNAs (miR-10b-3p and miR-34b-5p) showed to have potential biomarker of azoospermia.
Yao et al. [12]	60 non-obstructive azoospermic and 20 obstructive azoospermic	Spermatogonia, pachytene spermatocytes, and round spermatids	Illumina HiSeq 2000	A total of 396, 395, 378 miRNAs were differentially expressed in spermatogonia, spermatocyte and spermatid, respectively in non-obstructive azoospermic compared to obstructive azoospermic patients.
Piryaei et al. [70]	10 non-obstructive azoospermic and 8 obstructive azoospermic	Testicular biopsy	BGISEQ-500 platform	About 120 downregulated and 10 upregulation miRNAs in non-obstructive azoospermic cases compared to obstructive azoospermic. hsa-miR-449a, hsa-miR-34c-3p, hsa-miR-375, hsa-miR-517b-3p, hsa-miR-512-3p, hsa-miR-34c-5p, hsa-miR-520c-3p, hsa-miR-516b-5p, hsa-miR-1323, hsa-miR-34b-3p are top 10 downregulated miRNAs.
Wu et al. [8]	48 non-obstructive azoospermic and 48 fertile controls	Seminal plasma	qRT-PCR	miR-19b and let-7a found to be up-regulated in non-obstructive azoospermic patients compared to fertile controls.
Wu et al. [76]	100 non-obstructive azoospermic and 100 fertile controls	Seminal plasma	qRT-PCR	miR-141, miR-429 and miR-7-1-3p found to be significantly upregulated in non-obstructive azoospermic compared with fertile controls.
Barcelo et al. [77]	14 non-obstructive azoospermic, 13 obstructive azoospermic and 9 normozoospemic	Extracellular vesicles (sEVs) of seminal fluid	miRNA quantitative PCR panels	Sixty DE miRNAs in infertile patients compared with normozoosperic controls. miR-31-5p was identified as potential predictor of azoospermia.
Zhang et al. [78]	7 SCOS (Sertoli cell only syndrome), 6 hypospermatogenesis and 7 fertile controls	Seminal plasma	Illumina HiSeq 2000	78 miRNAs to be up-regulated and 132 miRNAs to be down-regulated in patients with SCOS (Sertoli cell only syndrome) pateints as compared to fertile controls, whereas 32 were up-regulated and 90 were down-regulated in patients with SA (spermatogenic arrest) patients in comparison to fertile controls.
Cito et al. [103]	14 non-obstructive azoospermic and 10 controls	Blood	qRT-PCR	Upregulated level of miR-20a-5p in non-obstructive azoospermic patients compared with fertile controls.
Naeimi et al. [104]	103 infertile men and 121 fertile control	Blood	qRT-PCR	miR-211 found to be downregulated in infertile men compared to the control.
Trzybulska et al. [105]	79 subfertile and 38 fertile controls	Serum	qRT-PCR	Serum miRNAs (miR-155-5p and miR-200c-3p potential biomarker for subfertility.
Liu et al. [9]	86 infertile and 86 fertile controls	Sperm	Microarray	About 56 downregulated miRNAs miR-574-5p, miR-297, miR-122, miR-1275, miR-373, miR-185, miR-193b were up-regulated and miR-100, miR-512-3p, miR-16, miR-19b, miR-23b and miR-26a were found in infertile males as compared to fertile males.
Abu-Halima et al. [89]	9 obstructive azoospermic, 9 azoospermic and 9 normozoospermic controls	Sperm	Microarray	Fifty miRNAs up-regulated and 27 miRNAs down-regulated in asthenozoospermic males. In oligoasthenozoospermic males, 42 miRNAs were up-regulated and 44 miRNAs down-regulated when compared with normozoospermic males.
Munoz et al. [90]	9 oligozoospermic and 7 normozoospermic fertile controls	Sperm	qRT-PCR	About 12 miRNAs were up-regulated (let-7b, -7c, -7 g, miR-21, -22, -30a, -148a, -221, -320a, -375, -423-3p, -423-5p) and 6 miRNAs were down-regulated (miR-25, -34b, -122, -152, -192, and 335) in oligozoopermic patients as compared to controls.
Mokánszki et al. [91]	10 oligozoospermic, 10 asthenozoospermic and 10 fertile controls	Sperm	qRT-PCR	Five miRNAs (let-7a, miR-7-1-3p, -141, -200a, -429) were significantly up-regulated and 3 miRNAs (miR-15b, miR34b, miR-122) were significantly down-regulated in the infertile group compared to the fertile group.
Salas-Huetos et al. [92]	10 asthenozoospermic, 10 teratozoospermic, 10 oligozoospermic and 10 fertile controls	Sperm	qRT-PCR	A total of 32, 19 and 18 differentially expressed miRNAs in asthenozoospermic, teratozoospermic and oligozoospermic men, respectively, as compared to controls
Salas-Huetos et al. [93]	8 normozoospermic infertile and 10 normozoospermic fertile	Sperm	qRT-PCR	Fifty-seven miRNAs were differentially expressed miRNA including 45 up- and 12 down-expressed miRNAs.
Abu-Halima et al. [94]	80 sub fertile and 90 fertile controls	Sperm and testicular biopsies.	qRT-PCR	hsa-miR-429 was found to be upregulated whereas hsa-miR-34b*, hsa-miR-34b, hsa-miR-34c-5p and hsa-miR-122 downregulated in spermatogenic disruption.
Vazquez et al. [95]	10 asthenozoospermic, 10 teratozoospermic, 10 oligozoospermic and 10 fertile controls	Sperm	qRT-PCR	hsa-miR-942-5p/hsa-miR-1208 and hsa-miR-34b-3p/hsa-miR-93-3p showed the greatest potential for detecting seminal alterations in infertile cohort.
Abhari et al. [98]	43 oligozoospermic and 43 fertile controls	Sperm	qRT-PCR	miR-21, miR-22 levels were significantly higher in oligozoospermic than those in normal controls.
Heidary et al. [99]	39 azoospermic and 35 fertile controls	Sperm	Solexa sequencing	Eighteen significantly altered miRNAs in azoospermic men in comparison to controls. Seven miRNAs (miR-1‐3p, miR‐197‐3p, miR‐296‐5p, and miR‐625‐3p, miR‐328‐3p, miR‐888‐3p and miR‐92b‐3p) validated by RT-PCR and miR-888-3p found to be significantly overexpressed in azoospermic cases in comparison with controls.
Liang et al. [101]	4 asthenozoospermic and 3 control males	Sperm	Microarray	Sixteen significantly differentially expressed miRNAs were identified. miR-6739 and miR34-5p were highly differentially expressed miRNAs.

### Testicular miRNA in male infertility

Testes consist of an impressive signature of miRNAs, which regulate several important biological pathways critical for the process of spermatogenesis. In the very first study, Barad et al. 2004 used microarray to identify miRNA signatures in five different tissues, i.e., liver, brain, thymus, placenta, and testis, and found abundant expressions of five miRNAs (let-7a, miR-10b, miR-134, miR-187 and miR-212) in the human testicular tissue [64]. After a few years, a microarray study was conducted by Lian et al. [65] on testicular biopsies of three non-obstructive azoospermic patients and two testicular biopsies were taken from the patients undergoing orchiectomy for prostrate carcinoma. They identified 154 down-regulated and 19 upregulated miRNAs in non-obstructive azoospermic (NOA) patients as compared to controls [65]. Abu-Halima et al. [66] conducted yet another microarray on testicular tissues of infertile men with different histopathologic patterns. Forty infertile patients were recruited, of which 12 were Sertoli cell only syndrome (SCOS), 12 had mixed atrophy (MA) and 16 had germ cell arrest at spermatocyte stage (GA) while sixteen individuals with normal spermatogenesis and normal structure of seminiferous tubules were recruited as controls. They found a total of 197, 68, and 46 differentially expressed miRNAs in SCO, MA and GA patients, respectively as compared to controls [66]. miR-449 family (miR-449a and miR-449b*) and miR-34 family (miR-34b*, miR-34b and miR-34c-5p) were found to be highly down-regulated in SCO, MA, GA groups as compared to controls. In normal spermatogenesis, these miRNAs are abundantly expressed in testis, suggesting that these two miRNA families are crucial for spermatogenesis [66]. Yang et al. [67] identified 770 miRNAs in normal testis tissue using NGS based technology for the first time. Another group of scientists reported the association of miR-302 cluster, miR-21, miR-29a and miR-367 with testicular germ cell tumor cases [60].

**Fig. 3:**
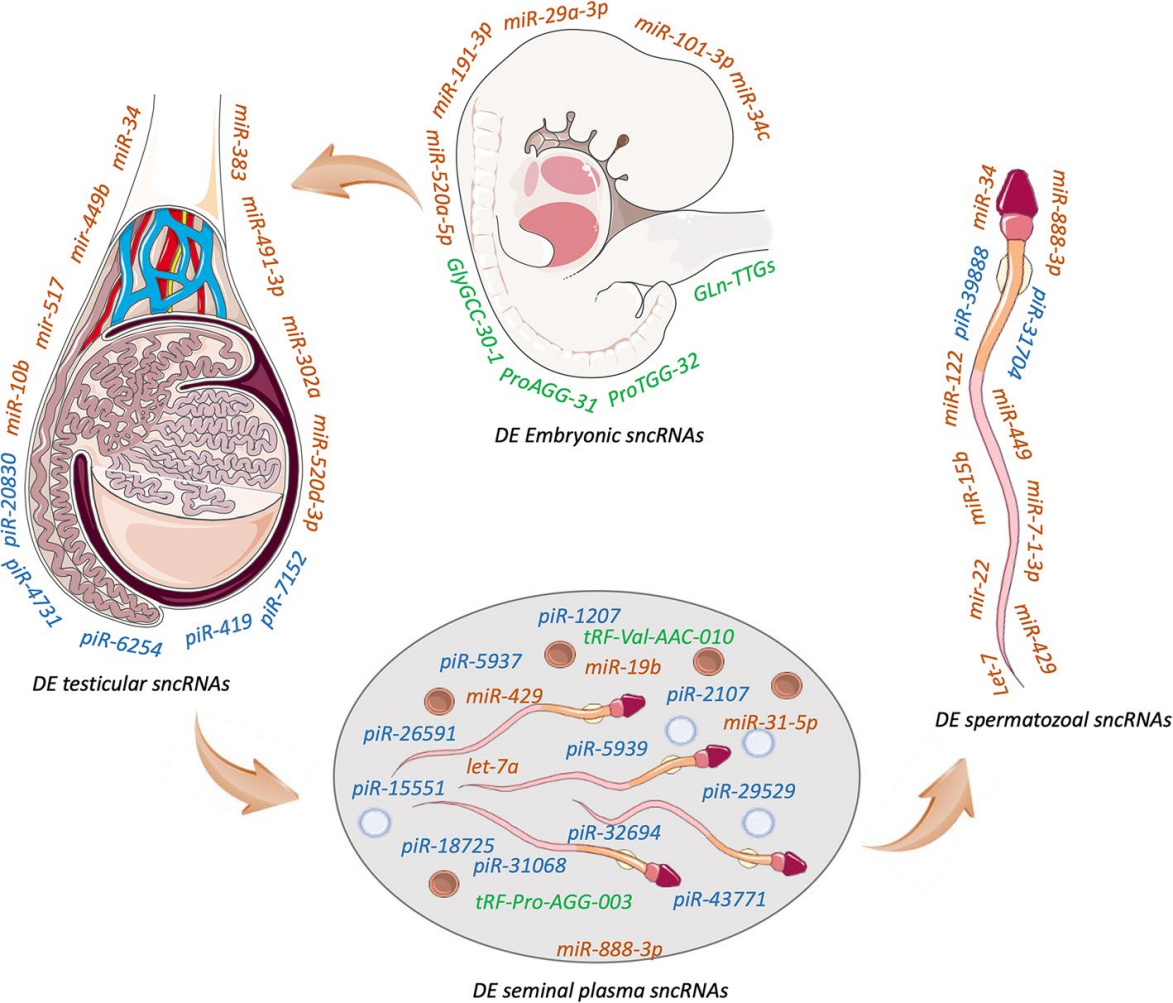
Differentially expressed small non-coding RNAs in primordial germ cells (PGCs), testis, seminal plasma, and sperm of infertile men. Parts of the figure were drawn using pictures from Servier Medical Art. Servier Medical Art by Servier is licensed under a Creative Commons Attribution 3.0 Unported License (https://creativecommons.org/licenses/by/3.0/ accessed on 15 January 2023)

Noveski et al. [68] conducted microarray analysis to identify miRNA expression in 27 hypospermatogenesis (HS) men, three hypospermatogenesis men with AZFc deletions, eight SCO, two MA, eight testicular atrophy/fibrosis/hyalinization (TFH) and eight 46,XXY men as compared to 18 obstructive azoospermic men recruited as controls. They identified six miRNAs to be down-regulated in patients with AZFc deletions, 27 miRNAs to be up-regulated and 3 down-regulated in MA patients, 32 miRNAs to be up-regulated and 20 miRNAs to be down-regulated in SCO patients as compared to controls. Five of the promising miRNAs (miR-34b, miR-449b, miR-517c, miR-181c, and miR-605) were validated by qRT-PCR. In another microarray study, Zhang et al. [69] identified 129 miRNAs to be differentially expressed in testicular tissues of NOA patients as compared to controls. Eight miRNAs were selected for validation by qRT-PCR, of which four were up-regulated (miR-370-3p, miR-10b-3p, miR-539-5p, miR-22-5p) and four were down-regulated (miR-34b-5p, miR-31-5p, miR-516b-5p and miR-122-5p) [69]. Apart from elucidating the miRNA expression in infertile testicular tissues, Yao et al. [12] conducted next generation sequencing to identify miRNA expression in isolated spermatogonia, pachytene spermatocytes and round spermatids from 60 NOA patients and 20 controls using STA-PUT. On further comparing the miRNA expression profile, they found a total of 396 DE miRNAs between spermatogonia of OA and NOA groups (88 miRNAs up-regulated and 308 miRNAs down-regulated), 395 DE miRNAs between pachytene spermatocytes of OA and NOA groups (97 miRNAs up-regulated and 298 miRNAs down-regulated) and 378 DE miRNAs between round spermatids of OA and NOA groups (64 miRNAs up-regulated and 314 miRNAs down-regulated) [12]. A recent study by Piryaei et al. [70] showed downregulation of 120 miRNAs and upregulation of 10 miRNAs in NOA cases when compared to OA. The top 10 downregulated miRNAs included hsa-miR-449a, hsa-miR-34c-3p, hsa-miR-375, hsa-miR-517b-3p, hsa-miR-512-3p, hsa-miR-34c-5p, hsa-miR-520c-3p, hsa-miR-516b-5p, hsa-miR-1323, hsa-miR-34b-3p [70]. Research done so far has concluded a significant link between altered expression of miR-34/449, let-7, miR-517 and spermatogenic disruption, leading to infertility in male (Table 1) (Fig. 3).

### Epididymal miRNAs

Epididymis has three distinct regions: caput, corpus and cauda. During sperm maturation, spermatozoa pass through the epididymis to acquire motility and fertilizing capability. In epididymis, sperm come in contact with different repertoires such as proteins and transcripts, which are released from the epithelium of epididymis via epididymosomes. It has been reported that sperm miRNA signatures change as they travel along the epididymis. Belleannѐe et al. [71] performed microarray in human epididymis from three donors to identify miRNA expression pattern in the epididymis of healthy males. They found 281, 282 and 289 miRNAs to be expressed in the caput, corpus and cauda region of epididymis, respectively, with 35 miRNAs showing differential expression across these segments of epididymis. Further, they found that five members of the miR-888 cluster (miR-890, miR-891a, miR-891b, miR-892a, and miR-892b) were abundantly expressed in caput and the corpus region, suggesting their role in the latter stages of sperm maturation [71]. In order to understand the role of miRNAs in epididymis during post-natal development, Zhang et al. [72] performed miRNA profiling in the epididymis of a new-born, 25 years old, and 75 years old males and found that epididymis of new born expressed more number of miRNAs whereas epididymis of 25 and 75 years old males expressed a few miRNAs, with 127 miRNAs exclusively expressed in the new-born epididymis whereas only 3 and 2 were exclusively expressed in the epididymis of 25 and 75 years old males, respectively. There were 106 miRNAs which were ubiquitously expressed in all the three ages, including let-7a, b, c, d, f, miR-125a, miR-125b, miR-143, miR-23b, miR-26a and rno-miR-347 [72]. In a case-control study, miRNA expression in epididymis of three control and three vasectomized patients was identified using microarray [71]. They identified a total of 336 and 394 miRNAs in the epididymis of vasectomized and control donors, respectively. Further, they identified four (miR-126, miR-129, miR-326 and miR-16), two (miR-1826 and miR-18a) and nine miRNAs (miR-941, miR-548b, miR-1826, miR-1281, miR-1825, miR-548c, miR-1278, miR-421 and miR-135a) that differed in expressions in caput, cauda and corpus, respectively after vasectomy. Additionally, they found a total of 22 miRNAs were down-regulated and 8 miRNAs were up-regulated in the epididymis of vasectomized patients as compared to controls [71]. Studies done so far have shown loading of 115 miRNA RNAs on spermatozoa and shedding of about 113 small RNAs from spermatozoa during the epididymal maturation process [73].

Recent investigations on the effect of stress on male fertility and transgenerational inheritance have shown a critical role of epididymis in altering the small RNA profile of spermatozoa, which may lead to a change in transcriptome of the zygote [74]. miR-31-5p was reported to be majorly altered miRNA along with miR-155-5p, miR-878-5p, and miR-34c-5p in cauda epididymosomes of adult males exposed to postnatal stress [74]. A number of other sperm-borne small RNAs have been shown to alter upon exposure to high fat diet, glucose intolerance, stress, resilience, and other endocrine disruptors [75]. These studies have provided evidences that these epididymis miRNAs may mediate transgenerational changes aimed at betterment of species.

### Seminal plasma miRNAs

Seminal plasma is constituted by the secretions of the testis, seminal vesicles, and prostate. A number of small RNAs leach out from the testicular cells or accessory glands to make their way into the seminal plasma. MicroRNAs are abundant and stable in seminal plasma, which can be exploited as biomarkers of fertility.

Seminal plasma has a large number of cell free small RNAs, which may originate in the testis, epididymis or male accessory glands. These small RNAs are often in extracellular vesicles, which have unknown significance. Till date, over 21,000 numbers of small RNAs have been reported in seminal plasma. While their biological significance remains hitherto unknown, their utilization as the markers of fertility and infertility has been tried by a number of studies. Extracellular small RNAs have particularly been interrogated in azoospermia cases where there are no sperm in the ejaculate.

Wu et al. [8] performed qRT-PCR on the seminal plasma of 48 NOA patients and 48 fertile controls and identified miR-19b and let-7a to be differentially up-regulated in NOA patients compared to fertile controls [8]. The same research group later performed TaqMan low-density arrays (TLDA) analysis to identify differential miRNA expression in 20 NOA patients and 20 fertile controls [76]. Two independent validations led to the identification of three miRNAs (miR-141, miR-429 and let-7-1-3p) that were significantly upregulated in NOA patients as compared to fertile controls. Further, the ROC curve analysis showed that these miRNAs could be used as semen-based biomarkers for diagnosis of NOA [76].

A few studies have identified altered miRNA expressions in the extracellular micro-vesicles present in the seminal plasma (Table 1). Barcelo et al. [77] undertook miRNA profiling analysis using qRT-PCR panels in 14 infertile azoospermic patients with spermatogenic failure and 13 obstructive azoospermic individuals with conserved spermatogenesis. They found that miR-31-5p could be useful in distinguishing between OA patients with conserved spermatogenesis from NOA patients with failed spermatogenesis. Additionally, they showed that the combined expression values of miR-539-3p and miR-941 could be useful in determining the presence of sperm in patients with severe spermatogenic disorders [77].

Recently, Zhang et al. [78] performed next generation sequencing to identify differentially expressed miRNAs in the seminal plasma of patients with different histopathologic patterns (7 Sertoli cell only-syndrome, 6 hypospermatogenesis/spermatogenic arrest and 7 normal fertile males). They found 78 miRNAs to be up-regulated and 132 miRNAs to be down-regulated in patients with SCOS as compared to fertile controls, whereas 32 were up-regulated and 90 were down-regulated in patients with SA in comparison to fertile controls. The expression level of miR-34c-5p was significantly down-regulated and hsa-141-3p was upregulated in the seminal plasma of NOA patients as compared to controls. Furthermore, they found that miR-34c-5p expression was significantly lower in the seminal plasma of SCOS patients as compared to patients with SA, suggesting that miR-34c-5p could be used as non-invasive biomarker to distinguish between patients with SCOS and patients with SA, which in turn could provide information regarding the chances of sperm retrieval from the testis [78]. In conclusion, evidence so far suggests the potential of seminal plasma miRNAs, miR-34c-5p, hsa-141-3p and let-7 as biomarkers to detect male infertility or the presence of spermatozoa in the testicular tissue of non-obstructive azoospermic patients (Fig. 3).

### Sperm miRNAs

Since the discovery of small RNAs in spermatozoa, researchers have delved into understanding the sperm RNA profile [79, 80]. Advances in sequencing technologies have encouraged researchers to investigate the microRNA profile of several types of cells, including spermatozoa, in detail, and eventually a plethora of miRNAs unique to spermatozoa have been identified [64, 79]. Initially, Krawetz and their colleagues revealed the presence of multiple classes of small RNAs in human spermatozoa using high-throughput sequencing [81]. They found that a healthy human sperm consists of around 7%, 17% and 65% of microRNA, piRNAs, and repeat associated small RNAs, respectively [81]. miRNAs in mammalian spermatozoa were systematically investigated and 951 known miRNAs and 8 novel, highly expressed miRNA candidates, were identified in bovine sperm [82]. Similarly, Salas- Huetos et al. [83] used qRT-PCR (TaqMan arrays) to identify miRNA signature in sperm samples from 10 fertile individuals. They found that 221 miRNAs were consistently expressed in all the individuals. Further, they found that hsa-miR-34b-3p as the most abundantly expressed miRNA and hsa-miR-663b to be the most stable miRNA. Investigation of miRNA expression prolife in two fertile donors by Pantano et al. [84] identified miR-1246 to be the most abundant miRNAs among total of 200 sperm miRNAs [84]. Studies so far indicate normal human spermatozoa to harbour about 24,000 sncRNAs small miRNAs; however, a proper study comparing miRNA profile across a good number of normozoospermic fertile individuals remains to be undertaken to identify normal human sncRNAome.

Spermatozoa carry a plethora of small and long non-coding RNAs, which were initially thought to be the remnants of past activity and considered to be functionally inert. However, a few studies have proven their functional importance in early embryonic development [85, 86]. Some of these RNAs have also been considered to be good candidates for investigating successful spermatogenesis on the basis of their testicular origin [19, 87]. Sperm-borne small RNAs have been found to be particularly interesting for their variations with health, diet, environmental exposures and other variables, and have gained special attention in transgenerational inheritance [88].

Irrespective of their testicular or extra-testicular origin and their functions, sperm RNAs have been proposed to serve as excellent markers of male fertility. Several studies have been conducted to identify miRNA signatures of fertility (Table 1) [9, 83, 89–91]. In the very first study, Liu et al. 2012 undertook microarray to identify altered miRNAs in semen samples from 86 infertile and 86 fertile males. They identified 56 miRNAs that were differentially expressed between the two groups [9]. Further, they validated their microarray data by qRT-PCR and found that miR-574-5p, miR-297, miR-122, miR-1275, miR-373, miR-185, miR-193b were up-regulated and miR-100, miR-512-3p, miR-16, miR-19b, miR-23b and miR-26a were down-regulated in infertile males as compared to fertile males [9].

Abu-Halima et al. [89] performed microarray to identify altered miRNAs in the sperm samples from 9 oligoasthenozoospermic, 9 asthenozoospermic patients and 9 fertile controls. They found that the highest altered miRNAs in asthenozoospermic samples were miR-34b, miR-122 and miR-1973, whereas miR-34b, miR-34b*, miR-15b, miR-34c- 5p, miR-122, miR-449a, miR-1973, miR-16, and miR-19a were altered in oligoasthenozoospermic patients as compared to controls [89]. Similarly, Munoz et al. [90] used qRT-PCR to analyze 23 miRNAs in sperm samples from 9 oligozoospermic infertile and 7 normozoospermic fertile males, and found that 12 miRNAs were up-regulated (let-7b, -7c, -7 g, miR-21, -22, -30a, -148a, -221, -320a, -375, -423-3p, -423-5p) and 6 miRNAs were down-regulated (miR-25, -34b, -122, -152, -192, and 335) in oligozoopermic patients as compared to controls. In the same year, Salas-Huetos et al. [92] analyzed 736 miRNAs by qRT-PCR in 10 oligozoospermic, 10 asthenozoospermic, 10 teratozoospermic infertile and 10 normozoospermic fertile sperm samples. They identified 32, 19 and 18 differentially expressed miRNAs in asthenozoospermic, teratozoospermic and oligozoospermic men, respectively, as compared to controls. An year later, the same group analysed 736 miRNAs in sperm of 8 normozoospermic infertile and 10 normozoospermic fertile males by qRT-PCR [93]. They identified 567 miRNAs which were differentially expressed between normozoospermic infertile and fertile groups. Recently, Mokánszki et al. [91] analysed 11 miRNAs in sperm samples from infertile (10 oligozoospermic and 10 asthenozoospermic) and 10 normozoospermic fertile samples by qRT-PCR [91]. They identified that 5 miRNAs (let-7a, miR-7-1-3p, -141, -200a, -429) were significantly up-regulated and 3 miRNAs (miR-15b, miR34b, miR-122) were significantly down-regulated in the infertile group (oligozoospermia and asthenozoospermia) as compared to the fertile group. In another study, Abu-Halima et al. [94] suggested a panel of five miRNAs (hsa-miR-34b*, hsa-miR-34b, hsa-miR-34c-5p, hsa-miR-429, and hsa-miR-122) to have the potential to be used as non-invasive biomarkers to diagnose patients with subfertility [94]. Similarly, another group identified miRNA pairs (hsa-miR-942-5p/hsa-miR-1208 and hsa-miR-34b-3p/hsa-miR-93-3p) that could be used as potential biomarkers of male infertility [95].

Recently, Dorostghoal et al. [96] reported that miR-26a-5p was down-regulated in sperm of normozoospermic infertile patients as compared to controls, whereas its target PTEN was up-regulated in sperm of normozoospermic infertile patients. ROC (receiver operating characteristic curve) analysis suggested that miR-26a-5p had potential to distinguish between fertile and unexplained infertile men [96]. In another study, the level of miR-23a/b-3p was found to be significantly upregulated in semen sample of oligoasthenozoospermic men as compared to controls. In-silico target prediction of miR-23a/b-3p identified different sperm proteins (PFKFB4, HMMR, SPATA6, and TEX15) as direct targets and found to be downregulated in infertile semen samples. Sperm count, motility, and morphology were also found to be negatively correlated with miR-23a/b-3p [97].

Abhari et al. [98] found an inverse association of miR-21 and miR-22 with estrogen receptor beta (ERß) using Real-Time PCR technique. The expression levels of hsa-miR-21 and hsa-miR-22 in 43 oligospermic infertile males (*n* = 43) were compared to 43 age-matched healthy controls. Compared to fertile control males, hsa-miR-21 and hsa-miR-22 were found to be downregulated and the level of ERß was significantly upregulated compared to normal control males [98]. This indirect regulation of these two miRNAs with estrogen receptor indicates their possible diagnostic value in male infertility. Compared to fertile men, overexpression of miR-888 in asthenozoospermic patients was detected along with other 17 significantly differentially expressed miRNAs. Taking the evidences from previous studies, Heidary et al. [99] showed a potential role of miR-888 as a diagnostic marker for asthenozoospermia [99].

Our previous study aimed at the identification of miRNAs as potential biomarkers of male infertility and sperm quality. A total of 41 oligo/oligoasthenozoospermic infertile, 40 asthenozoospermic infertile, 40 normozoospermic infertile, and 40 normozoospermic fertile samples were collected to compare the levels of seven miRNAs (hsa-let-7a-5p, hsa-miR-9-3p, hsa-miR-22-5p, hsa-miR-30b-5p, hsa-miR-103-3p, hsa-miR-122-5p and hsa-miR-335-5p) selected upon literature search. Receiver operating characteristic (ROC) curve analysis and validation by RT-PCR detected three (hsa-mir-9-3p, hsa-miR-30b-5p and hsa-miR-122-5p) miRNAs, which showed a close association with male infertility and can be considered as potential biomarkers of male infertility [100]. MiRNA profiling in 4 asthenozoospermic patients and 3 healthy control men by Liang et al. [101] showed 16 significantly differentially expressed miRNAs, of which 13 were validated by qRT-PCR, which included eight novel miRNAs (miR-5000-3p, miR-4289, miR-6514-3p, miR-6882-5p and miR-6739-5p, miR-135a-5p, miR-509-3p and miR-196b-5p) [101].

In a recent study, it was reported that miR-34 family was downregulated in sperm of infertile patients as compared to controls and also found that the promoter of miR-34b,c was hyper-methylated in infertile patients (highest methylation in asthenoteratospermia and oligoasthenoteratozoospermia), suggesting that the down-regulation of the miR-34 family is due to hyper-methylation of their promoter regions [102]. The association between dysregulated sperm miRNAs and male infertility clearly depicts the contribution of sperm miRNAs in regulating spermatogenesis (Fig. 3). Above mentioned evidences from several studies showed that miRNAs can be considered as potential biomarkers of male infertility.

### Blood-borne miRNAs in male infertility

An investigation of the potential of human blood plasma miRNAs in non-obstructive azoospermia patients showed a positive correlation of upregulated level of miR-20a-5p with follicle stimulating hormone (FSH) and luteinizing hormone (LH) and an inverse correlation with serum total testosterone (TT) [103]. Further investigations on blood plasma samples from 103 infertile men including, non-obstructive azoospermia (NOA) or severe oligozoospermia (SO) cases, and 121 fertile men showed a significant role of miR-211 as a predictive factor in blood plasma of infertile men (Table 1). The level of miR-211 was significantly downregulated in infertile cases, indicating that miR-211 was associated with sperm parameters and can be further explored in male infertility [104]. Trzybulska et al. [105] studied the link between metabolic disorder related serum miRNAs (miR-155-5p, miR-122-5p, miR-200a-3p, and miR-200c-3p) and male subfertility. However, no direct association of dysregulated miRNAs in sub-fertile cases was detected with metabolic syndrome (MetS) parameters [105].

### miRNA, single nucleotide polymorphisms (SNP), and male infertility

Like protein coding genes, genetic polymorphisms in the miRNA coding genes may affect their functions by disrupting miRNA-mediated regulation of protein-coding genes. The expression of several genes gets severely affected due to the loss or gain of function mutations in miRNA genes. miRNA function can be affected by polymorphisms not only in the miRNA coding sequences, but also by polymorphisms within miRNA target sequences or in the genes involved in miRNA biogenesis. There is a different terminology for polymorphisms in the miRNA-coding genes or in the miRNA-binding sites. miR-SNP refers to the variations in the miRNA gene sequence, whereas miR-TS-SNP refers to the variations in the miRNA target site.

Zhang et al. [106] were the first to report the association between SNPs in the miRNA binding site and male infertility [106]. They undertook 140 mammalian spermatogenesis-related genes and analysed all SNPs in the 3’UTR of these genes and found that certain SNPs were present in the miRNA binding sites. Out of 140 genes, 6 SNPs in the 3’UTR of the *CYP19, SERPINA5, CGA, CPEB1* and *CPEB2* genes were further analysed in patients with idiopathic azoospermia and severe oligozoospermia and fertile controls by genotyping. It was found that A > T substitution in the binding site of miR-1302 in the *CGA *gene was associated more strongly with idiopathic male infertility. Luciferase assay confirmed that the change from A > T in 3’UTR of the *CGA* gene hindered the binding of miR-1302 [106]. In a similar study, Lu et al. [107] reported that T > C polymorphism (rs11614913) in the hsa-miR-196a-2 gene was associated with idiopathic male infertility. Further, they found that genotype CC (rs11614913) was significantly associated with increased expression of hsa-miR-196a-5p in the seminal plasma of infertile patients. In addition to this, they found increased apoptosis upon GC2 treatment with hsa-miR-196a-2 mimic and decreased apoptosis upon treatment with hsa-miR-196a-2 inhibitor [107]. Similar to the above, polymorphisms in the *DROSHA* and *DICER* genes could affect the actions of miRNAs, making them good candidates for several human diseases. Qin et al. [108] observed seven SNPs, out of which four (rs10719, rs2291109, rs17409893, and rs642321) were present in the *DROSHA* gene and three (rs13078, rs1057035, and rs12323635) were present in the *DICER* gene. Real time PCR showed that two SNPs (rs10719, rs642321) in the *DROSHA* gene and one SNP (rs12323635) in the *DICER* gene were associated with male idiopathic infertility in Han Chinese population [108]. Ay et al. [109] analyzed six SNPs in six different genes i.e. *DROSHA* (rs10719), *DICER* (rs13078), *DGCR8* (rs1640299), *XPO5* (rs11077), *RAN* (rs14035) and *GEMIN3* (*DDX20*, rs197388) in idiopathic azoospermia, and observed a significant association of the AA genotype in the *GEMIN3* gene with idiopathic azoospermia. Evidence from other animal studies also suggests that polymorphisms in the miRNA machinery may lead to compromised fertility. Variations in miRNA seed regions may lead to decreased fertility and reproduction in economically important animals [109]. Similarly, Huang et al. [110] showed that a polymorphism in the 3’UTR of the *PCK1* gene disrupted miR-26a binding site and showed association with fertility in bulls. Further, they showed that polymorphism in *PCK1* does not affect *PCK1* expression, but was related to sperm motility, elevated ejaculation volume, and reduced values of composite index and calving ease.

### miRNAs in post-fertilization development

Germ-line *Drosha* and *Dicer* conditional knockout mice resulted in generation of gametes (sperm and ovary), which were partially deficient in miRNAs and endo-siRNAs [19, 111]. It was found that sperm from these mice were able to fertilize the egg, but the embryos showed developmental defects [19]. Later on, injecting small RNAs isolated from the wild-type sperm rescued these embryos, suggesting the importance of small RNAs in post-embryonic development. Recently, it was found that sperm miR-191 is associated with early embryonic development in humans [112]. High expression of miR-191 in sperm was found to correlate with high fertilization rate (FR), effective embryo rate (EER) and high-quality embryo rate (HQER) [112]. Therefore, miR-191 could be used to predict achieving high-quality embryo rate [112]. In another study, small RNA sequencing was undertaken to figure out the correlation of sperm-borne small RNAs in giving rise to high rate of good quality embryos (H-GQE) and low rate of good quality embryos (L-GQE) [29]. It was found that three miRNAs (miR-132-3p, miR-191-3p, and miR-520a-5p) were down-regulated and two miRNAs (miR-101-3p and miR-29a-3p) were up-regulated in the L-GQE group, suggesting that these miRNAs might play an important role in early embryonic development [29].

### Endogenous siRNAs in male germ cells and infertility

Endo-siRNAs were first reported in murine oocytes and embryonic stem cells [28, 113, 114]. Later, out of curiosity, Song et al. [17] investigated and found that mouse spermatogenic cells also express endo-siRNAs and that these endo- siRNAs can also target hundreds of transcripts in the genome [17]. They are involved in genome protection by repressing transposable elements and regulating gene expression by chromatin remodelling. siRNAs are double-stranded RNAs, which are processed by Dicer and loaded onto Ago2 to perform cleavage of target mRNAs by perfect base pairing. siRNAs have been widely used in gene expression studies in-vivo and in-vitro (Fig. 1) [115]. Han et al. [116] reported 26 nucleotides long endo-siRNAs, named as 26G RNAs, generated by germ cells in *Caenorhabditis elegance*. 26G RNA Class I was found to regulate the development of spermatozoa by regulating the expression of genes during spermatogenesis. On the other hand, class II 26G might play a role in zygotic development [116]. In yeast and worms, double-stranded RNA precursors are produced by RNA-dependent RNA polymerase (RdRP), followed by processing by Dicer [117, 118], while in mammals and flies RdRP is not present; therefore, naturally occurring dsRNAs like hairpin-dsRNAs, trans-antisense transcript-derived dsRNAs, and cis-antisense transcript-derived dsRNAs, are considered to be endo-siRNA precursors for Dicer processing [18]. As the microprocessor complex DGCR8/DROSHA is required for miRNA precursor processing but not for endo-siRNA synthesis, Zimmermann et al. [119] used this comparison to derive the essential role of endogenous siRNAs in progression of spermatogenesis [119].

It has been found that half of the siRNA population is expressed during a particular stage in spermatogenesis, whereas the remaining half is expressed ubiquitously throughout spermatogenesis [120], which implies their roles in housekeeping activities as well as stage-specific regulation of gene expression in the testis. However, siRNAs have not been investigated for their association with infertility.

### piRNAs in male germ cells and infertility

piRNA is a class of small RNAs that interacts with the Piwi clade of Argonaute proteins; therefore, called as piwi interacting RNAs (piRNAs). Piwi clade consists of HILI, HIWI1, HIWI2 and HIWI3 proteins in humans, Aubergine (Aub) and Ago3 in flies, and MILI, MIWI, MIWI2 in mice. piRNAs are processed from long single stranded precursor RNAs transcribed from piRNA clusters. A-MYB is the only transcriptional factor known to be involved in the transcriptional regulation of pachytene piRNA precursors. The biogenesis of piRNAs from these long precursor RNAs is called primary piRNA biogenesis (Fig. 1). MOV10L1, a RNA helicase and MitoPLD/Zucchini, an endonuclease, process the longer transcripts and generate shorter RNAs, termed as piRNA intermediates. piRNA intermediates, with 5’monophosphate group, get loaded onto the MIWI proteins and get processed from the 3’end by exonuclease activity of PNLDC1 and 2’O methylated on their 3’end by HEN1 [121]. In the secondary biogenesis pathway, primary piRNA directs and associated MILI/MIWI protein cleaves the target transcript and generates 5’monophosphate, which further loads MIWI/MILI and generates secondary piRNA with 10nt complementarity to the primary piRNA [121].

piRNA  is a very diverse class of small RNAs, comprising of more than 1.5 million distinct sequences, which map to hundreds of genomic clusters [122–124]. In mammals, piRNAs are classified mainly into two types according to their expression surge in specific stages of spermatogenesis i.e., pre-pachytene piRNAs expressed in prospermatogonia and pachytene piRNAs expressed in pachytene spermatocytes. During spermatogenesis, MILI, MIWI and MIWI2 show dynamic expression, with stable expression of MILI from 12.5 prenatal stage to adulthood and limited expression of MIWI2 from 15dpc to 3dpp stage and predominant expression of MIWI in the pachytene spermatocytes and round spermatids [27]. In addition to distinct expression pattern of MIWI proteins, they are also known to associate with distinct length of piRNAs; for example, MILI associates with 26–27 nt long piRNAs, and MIWI associates with 29–30 nt long piRNAs [125].

The main function of piRNAs is to silence the transposable elements (TEs) in the germ line. In flies, Aravin and colleagues reported that a class of relatively longer small RNAs (~ 25–30 nt) were involved in the silencing of repetitive/transposable elements [126]. In animal germ cells, it has been reported that PIWI/piRNA machinery silences transposable elements at both the epigenetic and post-transcriptional levels [127–129]. The pre-pachytene piRNA due to their repeat derived nature are known to regulate de novo methylation and TE expression during the methylation re-writing phase of embryonic development [27]. The mechanistic investigations revealed MIWI2 interacting protein SPOCD1 as an essential executor of de novo methylation [130]. The SPOCD1 mutant mice were infertile and have increased expression of LINEs (Long interspersed nuclear element) and IAPs (intracisternal A particles) [130]. Whole genome sequencing in mutant mice revealed demethylation in IAPEy and young LINE families [130]. The pachytene piRNAs originate from intergenic non-coding regions and 3’ UTR regions of protein coding transcripts, pseudogenes and a few from repeat regions, which suggests their roles beyond TE silencing in this phase. Pachytene piRNAs have been shown to target mRNAs and lncRNAs via PTGS (post transcriptional gene silencing) mechanism [28]. This notion was further supported by HENMT1 mutant mice which regulates 3’O methylation of piRNAs and their stability. The HENMT1 deletion caused global loss of piRNAs and dysregulation of spermiogenic genes [131]. The mechanism of mRNA regulation through piRNA is independent of slicer activity of MIWI and dependent upon deadenylation of mRNA by CAF1 with subsequent degradation [16]. Another finding identified such pairs where transcript level remained the same with piRNA alteration, which elucidated a new mechanism of action of piRNA by translational activation of genes required for spermatid development [132]. In addition to the Piwi proteins, Tudor domain protein family also plays a major role in the piRNA pathway. These proteins have been identified in mouse and *Drosophila* and play a major role in piRNA accumulation and target regulation by their interaction with Piwi proteins [133]. The exact role of piRNAs in the process of spermatogenesis and their importance in embryonic development, if any, is not yet clear. There is still a lot to discover and understand in this field.

The knockout studies on PIWI pathway genes have majorly observed meiotic arrest phenotype and a few showed post-meiotic arrest [134]. Mutations in the piRNA biogenesis genes have been linked to non-obstructive azoospermia and severe oligozoospermia. For example, biallelic loss of function mutations in the *FKBP6* gene (c.508_529dup, p.Phe177CysfsTer20) showed an association with extreme oligozoospermia condition [135]. Similarly, biallelic stop gain mutation p.R452Ter (rs200629089), compound heterozygous mutations p.M259T (rs141903829) and p.L35PfsTer3 (rs754159168), a novel biallelic missense variant p.P84S, a splice acceptor site variant c.607-2 A > T [136] and p.R47G, p.E381K, p.R476W [137, 138] mutations in the *PNLDC1* gene [139], and loss of function mutations (T110Nfs*30 and Y230X) in the *TDRD7* gene [140] have been reported in non-obstructive azoospermia. In addition to this, *HIWI2* rs508485 and *HIWI3* rs11703684 polymorphisms have been associated with idiopathic non-obstructive azoospermia in an Iranian population [141]. Apart from the above, epigenetic alterations in piRNA biogenesis genes have also been reported in spermatogenic failure cases. *PIWIL2* and *TDRD1* promoter regions were hypermethylated in round spermatid maturation failure cases [142]. Further, the expression studies on Tudor domain containing proteins (TDRDs) have shown reduced levels of TDRD1, TDRD5, TDRD9 and TDRD12 in non-obstructive azoospermia [143].

Apart from genetic and expression studies on piRNA machinery elements, the small RNAs expression studies have also been undertaken in biopsy samples and seminal plasma of non-obstructive azoospermia cases (Table 2). A study on successful and unsuccessful TESE cases of NOA have shown dysregulation in piRNA expression with 951 downregulated piRNAs and 8 upregulated piRNAs. They have further validated 20 differentially expressed piRNAs (hsa-piR-20,830, hsa-piR-4731, hsa-piR-6254, hsa-piR-419, hsa-piR-7152, hsa-piR-7548, hsa-piR-14,195, hsa-piR-5026, hsa-piR-11,482, hsa-piR-17,765, hsa-piR-17,102, hsa-piR-4484, hsa-piR-17,260, hsa-piR-17,098, hsa-piR-20,511, hsa-piR-5802, hsa-piR-19,121, hsa-piR-2510, hsa-piR-4745, hsa-piR-11,873) using quantitative real time PCR, which can be further studied for their biomarker potential in micro-TESE application [144]. Recently Chen et al. [145] has identified 8 piRNAs correlated with sperm concentration and they were used for the evaluation of status of spermatogenesis in testis by detecting them in seminal plasma extracellular vesicles. They identified piR-61,927 holds the potential to be established as a predictor of micro-TESE outcome in NOA patients [145] Seminal plasma piRNA comparison has also revealed five potentially downregulated markers with high diagnostic potential (piR-31,068, piR-31,925, piR-43,771, piR-43,773 and piR-30,198) to distinguish infertile subjects from fertile ones [146] (Fig. 3). The piRNA expression studies on exosomes isolated from seminal plasma of asthenozoospermic patients reported downregulated piRNAs (piR-1207, piR-2107, piR-5937 and piR-5939) as the molecular biomarkers for male infertility [147]. Another study on seminal plasma of asthenozoospemic and normal healthy males identified 114 upregulated and 169 downregulated piRNAs, The top 10 upregulated piRNAs included piR-26,591, piR-32,694, piR-18,725, piR-16,782, piR-25,491, piR-5952, piR-18,611, piR-18,586, piR-15,811, piR-17,725 and top 10 downregulated piRNAs included piR-15,551, piR-29,529, piR-25,106, piR-2645, piR-9170, piR-2912, piR-23,203, piR-6148, piR-32,713, piR-29,155 [148].

The lone study that analyzed three sperm-borne piRNAs (piR-31,704, piR-39,888 and piR-40,349) in human infertility found that two piRNAs (piR-31,704 and piR-39,888) showed decreased expression in subnormal sperm concentration and a significant increase in the levels of piR-31,704, piR-39,888 and piR-40,349 in the spermatozoa with higher 2PN rate [149]. However, there was no correlation between the levels of these piRNAs with embryo early cleavage, good quality embryo, or pregnancy [149].

**Table 2:** Studies on differential expression of piRNAs in male infertility

**Study**	**Population type/size**	**Sample**	**Methodology**	**Inference**
Chen et al. [145]	8 non- obstructive azoospermic and 8 fertile controls	Extracellular vesicles piRNAs	Small RNA sequencing	piR-61,927 in seminal plasma EVs to predict the micro-TESE outcome in non- obstructive azoospermic patients.
Hong et al. [146]	118 asthenozoospermic, 93 azoospermic and 91 fertile controls	Seminal plasma	Small RNA sequencing and qRT-PCR	61 piRNAs showed 10-fold difference between infertile and fertile group; piR-31,068, piR-43,771, piR-31,925, piR-43,773, piR-30,198, piR-55,272, piR-30,841 and piR-30,495 validated using qRT-PCR.
Hong et al. [147]	10 asthenozoospermic and 10 normozoospermic	Seminal exosomes and sperm	qRT-PCR and small RNA sequencing	piR-1207, piR-2107, piR-5937 and piR-5939 were significantly reduced in exosomes from asthenozoospermia patients. 22 piRNAs were downregulated (<-1.5) in sperm sample of asthenozoospermic men when compared to normozoospermic men.
He et al. [148]	3 asthenozoospermic and 3 fertile controls	Seminal plasma	Small RNA sequencing	Top 10 upregulated piRNAs: piR-26,591, piR-32,694, piR-18,725, piR-16,782, piR-25,491, piR-5952, piR-18,611, piR-18,586, piR-15,811, piR-17,725; top 10 downregulated piRNAs piR-15,551, piR-29,529, piR-25,106, piR-2645, piR-9170, piR-2912, piR-23,203, piR-6148, piR-32,713, piR-29,155.
Cui et al. [149]	186 infertile patients and 40 fertile controls	Sperm	qRT-PCR	piR-31,704 and piR-39,888 were decreased in infertile group compared to control men.

### ts-RNAs in male germ cells and infertility

t-RNA is a class of non-coding RNA, which assists in decoding of mRNAs into proteins by transporting amino acids to the site of translation. Mature sperm being transcriptionally and translationally inactive do not require tRNAs and hence tRNAs are cleaved into small RNAs called as tRNA-derived small RNAs (ts-RNAs). These ts-RNA can be further divided into five groups based on their cleavage site or the region from which they are derived i.e., 5’-tsRNA halves, 3’-tsRNA halves, 5’-tsRNAs, 3’-tsRNAs, and anti-codon ts-RNAs [29]. 5’-tsRNA and 3’-tsRNA are highly similar across species as they are derived from the conserved mature tRNA sequences [150]. 5’-tsRNAs halves and 3’-tsRNAs halves are also referred as tiRNAs due to their stress induced characteristics [151]. 5’-tsRNAs are further divided into 3 types based on their cleavage site on parental tRNA i.e., tRF-5a (14–16 nt), tRF-5b (22–24 nt), tRF-5c (28–30 nt) [152].

tsRNAs not only regulate translation, but also regulate mRNA stability, stress response, and cell proliferation [153]. The very first study on tRNAs in mouse spermatozoa revealed that spermatozoa are enriched with a novel class of t-RNA derived small RNAs and termed them as mse-tsRNAs (mature-sperm-enriched tRNA-derived small RNAs) [154]. They discovered that mse-tsRNAs are divided into seven families, with family 1 (tRNA Glu) and family 2 (tRNA Gly) being the most prevalent ones. These abundantly expressed small RNAs originate from multiple sites on the genome, and most of the clusters are located on chromosomes 1, 8 and 13 (Fig. 1) [154]. Moreover, they showed that mse-tsRNAs were abundantly localized in the sperm head, suggesting their role in fertilization and early embryonic development [154].

Several small RNAs, such as miRNAs and tsRNAs are loaded on to spermatozoa during post-testicular maturation in the epididymis via epididymosomes [30, 73, 154, 155]. Approximately 70% of small RNAs in mature spermatozoa are tsRNAs [155]. Nixon et al. [150] revealed that sperm in the cauda region of mouse epididymis are abundantly rich in tsRNAs. Most of these tsRNAs were derived from the 5’ half of the mature tRNA molecules [150]. Sharma et al. [156] showed that there is a change in sperm RNA dynamics from piRNAs to tRNA fragments as spermatozoa transits from testis to the epididymis [151]. The tRNA derived small RNAs are transported to spermatozoa via vesicles called epididymosomes, which are released from the epididymal epithelium during maturation [148, 149, 151]. All the above studies suggest that tRNA fragments could have key role in fertilization and embryonic development. Han et al. [157] discovered the potential of seminal plasma extracellular vesicles tRF-Val-AAC-010 as a non-invasive biomarker for predicting the likelihood of sperm retrieval in NOA patients [157]. The studies investigating the tsRNAs linked to various contexts of infertility are summarized in Table 3.

tRNA-derived small RNAs have also been found to be involved in intergenerational and transgenerational/epigenetic inheritance of the environmental experiences/stressors and metabolic disorders [30, 31]. A population of tRNAs derived small RNAs produced from the 5’end of tRNAs was reported to be altered in sperm of high fat diet (HFD) fed male mice [30]. Injections of sperm tsRNAs from HFD males into the normal zygotes resulted in metabolic disorders in the F1 generation as well as changes in the expressions of the metabolic pathway genes in early embryo and the islets of the F1 generation [30]. Altered tsRNA expressions were also reported in mice with advanced age [31]. Injections of sperm tsRNAs from aged mice spermatozoa into zygotes resulted in anxiety like behavior in the F1 generation males [31].

Most of the studies investigating the roles of tsRNAs have been carried out in zygotes/embryos (Table 3) (Fig. 3). Chen et al. [158] reported that Gln-TTG–derived small RNAs (Gln-TTGs) were highly enriched in ejaculated spermatozoa of pigs [158]. Microinjections of antisense Gln-TTG tRNAs into the zygotes resulted in decreased rates of two-cell, four-cell and blastocyst embryos, suggesting that Gln-TTG RNAs are crucial for early cleavage stages of embryo development [158]. In another study, Hua et al. [29] reported 10 tsRNAs (GlyGCC-30-1, GlyGCC-30-2, ThrTGT-38, ThrTGT-39, GluTTC-23, ProAGG-32, ProTGG-32, ProAGG-31, AsnATT-20, and ArgCCG-33) to be differentially expressed between L-GQE (low rate of good quality embryos) and H-GQE (high rate of good quality embryos) [29]. In order to explore the role of this tsRNA in human embryo development, they injected antisense Gln-TTGs in tripronuclear (3PN) human zygotes and found that the development of zygotes to four cells, eight cells and blastocyst embryo stages were reduced as compared to untreated zygotes. Moreover, the overexpression of Gln-TTGs promoted embryo development, suggesting that the tsRNA Gln-TTGs may serve as a potential biomarker for assessing sperm and embryo quality in IVF clinics [159].

**Table 3:** Studies on differential expression of tsRNAs in infertility

**Study**	**Population type/size**	**Sample**	**Methodology**	**Inference**
Hua et al. [29]	87 normozoospermic infertile males undergoing IVF treatment	Sperm samples	Deep sequencing	Ten tsRNA [(Downregulated: GlyGCC-30-1, GlyGCC-30-2, ThrTGT-38, ThrTGT-39, and GluTTC-23). (Upregulated: ProAGG-32, ProTGG-32, ProAGG-31, AsnATT-20, ArgCCG-33)] were differentially expressed in High- and low-grade quality embryo.
Han et al. [157]	41 non-obstructive azoospermic, 15 obstructive azoospermic, 5 idiopathic oligospermic and 12 fertile controls	Seminal plasma extracellular vesicle	Sequencing and qRT-PCR	tRF-Val-AAC-010 and tRF-Pro-AGG-003 were found to be upregulated in non-obstructive azoospermic and oligozoospermic compared to healthy male. These can be the biomarker for diagnosis of NOA and idiopathic oligospermia patient.
Chen et al. [159]	Human/ pooled five each (low, average and good quality embryo)	Embryos	Single cell RNA-seq	tsRNA-GLn-TTGs are differentially expressed in high- and low-quality sperms and also regulate cleavage of 3PN embryo.

### Small RNA based gene therapy

Small RNA-guided post-transcriptional regulation of genes is a natural ubiquitous cellular mechanism. Target specificity and the ability to regulate the level of various genes makes them good candidates for use as molecular therapeutic agents. The mechanism of RNAi can be activated by exogenous short hairpin RNAs expressed via viral vectors, which are processed into small RNAs intracellularly or by directly transfecting the cells with synthetic siRNAs (small interfering RNAs). In the cytoplasm, siRNAs are loaded on the RISC, which cleaves one strand. The other strand performs RNAi by binding to its target mRNA bearing a complementary sequence.

Synthetic siRNAs have been reported to treat diseases in mice [160]. Synthetic small RNAs have been used to knockdown a gene of interest to elucidate its role in the cell [161]. It was earlier thought that gene knockdown by small RNAs is target specific, but later it became clear that off-targets effects are also seen as the small RNAs may also suppress other genes having homologous sequences. Therefore, the main challenges to use small RNAs as a therapeutic agent are to protect the oligonucleotides from RNase mediated degradation, to avoid off target effects and to diminish the adverse effects caused by immune reaction in response to the exogenous delivery. Nevertheless, chemical modifications produce phosphorothioate-containing oligonucleotides, methylphosphonate-containing oligonucleotides, boranophosphate-containing oligonucleotides, 2′-O-methyl-(2′-O-Me) or 2′-O-methoxyethyl oligonucleotides (2′-O-MOE), 2′-fluoro oligonucleotides (2′-F), locked nucleic acid (LNA) oligonucleotides, peptide nucleic acids (PNAs), phosphorodiamidate morpholino oligomers (PMOs), which are found to reduce the off-target effects. Nevertheless, the inhibition of a gene is dependent upon how these small RNAs are delivered into the cell. There are two major vehicles for gene delivery: viral and non-viral vectors. Though viral vectors are more efficient than the non-viral vectors, the latter are preferred because of safety concerns with viral vectors. The use of exosomes to deliver small RNA may serve as a promising vehicle. Exosomes are nano-sized, natural vesicles secreted by cells and one of their roles is to transport small RNAs between cells. Therefore, exosomes could overcome the problem with conventional small RNA delivery systems.

Since alterations in miRNAs and piRNAs expression profile are related to male infertility, their antagonists and mimics can be used as an approach for male contraception. Since, most of these miRNAs and piRNAs are testis-specific, there would be very few side effects. Since hundreds of small RNAs participate to make spermatogenesis possible, instead of targeting several of them, RNAi targeting miRNA biogenesis genes may provide new approach for male contraception in future as mutations in the miRNA biogenesis genes (like Dicer and Drosha) in male infertility have already been reported.

The first small interfering RNA (siRNA) drug, Patisiran was granted approval by the FDA in 2018. This drug was approved for a rare disease, hereditary transthyretin-mediated amyloidosis. It works by targeting and degrading the messenger RNA transcript for Transthyretin [162, 163] Although none of the miRNAs have been approved by FDA so far, yet a number of them are in clinical development or in phase 1 or 2 clinical trials [164]. Altered miRNA expression in different diseases can be reverted by using miRNA mimics or a gene coding for miRNA into viral constructs in diseases where they are down-regulated or by using miRNA inhibitors to block the expression of miRNA in diseases where they are up-regulated [165] Synlogic, Miragen, and Regulus Therapeutics are the biotech companies which are solely involved in improving the miRNA-related drug pipelines. Several miRNAs are in phase 1 and 2 of clinical trials while some have failed, resulting in termination of their studies. For example, MRX34, an miR-34 mimic drug for cancer, was halted in phase 1 trial by biotech company Synlogic for showing severe adverse events (SAE) in five patients experiencing immune responses [164]. Recently, a drug MRG-110 has been found to have potential clinical application in wound healing and heart failure [166]. This drug inhibits miR-92 and promotes the growth of new blood vessels around the wound area [167]. A biotech company, miRagen has announced its second phase 1 trial. Another antimiR drug, Cobomarsen (inhibitor of miR-155) is in phase 2 trial under miRagen [168] This drug is used for the treatment of T-cell lymphoma. Similarly, antimiR drug Miravirsen, which is an inhibitor of miR-122, is a potential drug used for the treatment of Hepatitis C virus (HCV) infections. Last year, a biopharma company, Regulus announced a miRNA drug RGLS5579, which is an inhibitor of miR-10b, to have therapeutic potential for the treatment of glioblastoma multiforme [169]. In a nutshell, a few potential tumor suppressor miRNA candidates have reached clinical trials, and there are few other, such as mR-200, miR-335, miR-126, miR-143/145 and the members of the let-7 miRNA family, which are currently under preclinical stage and will soon enter phase 1 clinical trials [170].

Testis may be a more suitable organ for gene therapy in comparison to other organs due to the presence of blood testis barrier. Due to restricted exchange between the blood supply and inter-testicular compartment, there would be less chances of side effects because of therapeutic small RNA reaching to other organs. Small RNA therapy in case of infertility would need direct injection in the testis; therefore, the concern of general toxicity may not be that significant.

### Conclusion and future perspective

Small non-coding RNAs (sncRNAs) have been investigated in the context of spermatogenesis, fertility, and infertility for more than a decade. The abundance and stability of sncRNAs provide a valuable opportunity for harnessing them as reliable biomarkers of fertility. Several studies have shown significantly altered expressions of sncRNAs in testicular tissue, spermatozoa, and seminal plasma of infertile men and identified a number of differentially expressed sncRNAs to correlate with infertility. Notable small RNAs showing differential expression in infertility include miR-122, miR-34 family, miR-10b, let-7 family, miR-151, miR-449 family, tsRNA-GLn-TTGs, tRF-Val-AAC-010, tRF-Pro-AGG-003, piR-61,927, piR-31,704, piR-39,888, piR-823, piR-1207, piR-2107, and piR-31,068, among others. The identification and characterization of specific small RNA signatures associated with infertility offer valuable insights into the mechanisms involved in spermatogenesis and may provide non-invasive diagnostic targets and therapeutic biomolecules for male infertility.

Compared to other sncRNAs, miRNAs have been extensively studied in male infertility. In-depth exploration of miRNAs has unveiled their crucial significance in the development of germ cells as well as their involvement in key processes such as sperm maturation, fertilization, and post-fertilization development. We compared miRNA data published across nine studies and identified three miRNAs (hsa-miR-9-3p, hsa-miR-30b-5p, and hsa-miR-122-5p) as biomarkers of sperm quality and fertility [100]. This list will grow with more studies pouring in relevant data. Moreover, studies focusing on piRNA cluster deletions have shown their crucial role in fertility, and piRNA differential expression in human infertility has been studied only in five studies. Despite being relatively new in the realm of sncRNAs exploration, tsRNAs have emerged as a promising area of study. Recent studies suggest that tsRNAs hold great potential as biomarkers of sperm quality and fertility; however, there are only three studies on tsRNAs in human male infertility. Similar to miRNAs, piRNAs and tsRNAs hold great promise for identifying novel biomarkers of sperm quality and fertility.

miRNAs have a prominent presence in the testis, epididymis, and mature spermatozoa, suggesting their diverse roles, including participation in the regulation of various germ cell developmental events in the testis. piRNAs are abundant in the testis and may participate in critical processes such as safeguarding genome integrity, regulating gene expression, and regulating transposable elements' activities. tsRNAs largely originate in the epididymis and get loaded on spermatozoa during maturation; therefore, they may have significant roles in post-fertilization development and may be critical elements in transgenerational inheritance and environmental impact on fertility.

Small RNAs also hold great therapeutic potential for the treatment of male infertility. While there is no study on infertility treatment yet, small RNAs have shown promise in treating other conditions. For example, a few miRNAs, including miR-34c, miR-122, and miR-10b, have entered clinical trials and are being tried for therapeutic purposes in diabetes and cancer. There are small RNAs that could be tried for similar applications in male infertility in the future. There are numerous unresolved questions regarding sncRNAs, and several sncRNAs remain to be identified in association with male infertility. To fully exploit the potential of small RNAs in understanding and unravelling the regulatory mechanisms of spermatogenesis, it is imperative to gain a deeper understanding of their intricate roles in spermatogenesis and fertility. With regard to their understanding and applications in infertility, we have touched only the tip of the iceberg, and much deeper inside remains elusive.

## Data Availability

Not applicable.

## Abbreviations

sncRNA: Small non-coding RNAs
miRNA: MicroRNA
siRNA: Small interfering RNA
piRNA: PIWI-Interacting RNAs
tsRNA: tRNA derived small RNAs
rsRNAs: rRNA derived small RNAs
PGCs: Primordial germ cells
TE: Transposable elements
RISC: RNA-induced silencing complex
AGO: Argonaute
ESC: Embryonic stem cells
SSCs: Spermatogonial stem cells
SCOS: Sertoli cell only syndrome
MA: Mixed atrophy
GA: Germ cell arrest at spermatocyte stage
DE: Differentially expressed
TLDA: TaqMan low-density arrays (TLDA)
ROC: Receiver operative curve
FR: Fertilization rate
EER: Effective embryo rate
H-QER: High-quality embryo rate
L-QER: LOW- quality embryo rate
TESE: Testicular sperm extraction
